# High-dimensional analysis of the aging immune system: Verification of age-associated differences in immune signaling responses in healthy donors

**DOI:** 10.1186/1479-5876-12-178

**Published:** 2014-06-21

**Authors:** Diane M Longo, Brent Louie, Jason Ptacek, Greg Friedland, Erik Evensen, Santosh Putta, Michelle Atallah, David Spellmeyer, Ena Wang, Zoltan Pos, Francesco M Marincola, Andrea Schaeffer, Suzanne Lukac, Radha Railkar, Chan R Beals, Alessandra Cesano, Leonidas N Carayannopoulos, Rachael E Hawtin

**Affiliations:** 1 Nodality, South San Francisco, CA 94080, USA; 2 Research Branch, Sidra Medical and Research Centre, Doha, Qatar; 3 Infectious Disease and Immunogenetics Section, Department of Transfusion Medicine, Clinical Center, and Center for Human Immunology, National Institutes of Health, Bethesda, MD 20892, USA; 4 MTA-SE “Lendület” Experimental and Translational Immunomics Research Group, Budapest H-1089, Hungary; 5 Merck & Co., Inc, Rahway, NJ 07065, USA

**Keywords:** Multi-parameter flow cytometry, Systems immunology, Aging, Immune signaling

## Abstract

**Background:**

Single-cell network profiling (SCNP) is a multiparametric flow cytometry-based approach that simultaneously measures evoked signaling in multiple cell subsets. Previously, using the SCNP approach, age-associated immune signaling responses were identified in a cohort of 60 healthy donors.

**Methods:**

In the current study, a high-dimensional analysis of intracellular signaling was performed by measuring 24 signaling nodes in 7 distinct immune cell subsets within PBMCs in an independent cohort of 174 healthy donors [144 elderly (>65 yrs); 30 young (25–40 yrs)].

**Results:**

Associations between age and 9 immune signaling responses identified in the previously published 60 donor cohort were confirmed in the current study. Furthermore, within the current study cohort, 48 additional immune signaling responses differed significantly between young and elderly donors. These associations spanned all profiled modulators and immune cell subsets.

**Conclusions:**

These results demonstrate that SCNP, a systems-based approach, can capture the complexity of the cellular mechanisms underlying immunological aging. Further, the confirmation of age associations in an independent donor cohort supports the use of SCNP as a tool for identifying reproducible predictive biomarkers in areas such as vaccine response and response to cancer immunotherapies.

## Background

Due to an age-related decline in the function of the immune system, the elderly are more susceptible to infectious diseases and less likely to mount a sufficient response to vaccination [1,2]. However, the mechanisms underlying immunosenescence are incompletely understood. While mouse models have helped to reveal age-associated defects in T cells [3], due to species-specific differences in immunological aging, scientific findings in mice do not always translate directly to humans [4]. Recent studies of age-related immunological defects in human samples have been highly focused on characterizing age-associated changes in T cells [5]. Thus, such studies are often performed on purified T cells or T cell subsets, which precludes the identification of age-associated changes in the multitude of immune cell types intentionally removed/depleted from the purified sample. Further, traditional immunological studies often utilize population-level approaches such as Western blots which fail to capture heterogeneity within the cell population/subpopulation under scrutiny. A systems biology approach capable of capturing the functional behavior of the multiple cell types that interact within the human immune system is necessary to gain a comprehensive understanding of the complex mechanisms responsible for immunological aging.

SCNP is a multiparametric flow-cytometry based analysis that can simultaneously measure both phenotypic surface markers and intracellular signaling proteins in response to extracellular modulation in multiple cell subtypes within heterogeneous populations, including PBMCs [6]. Thus, SCNP allows for a high-dimensional analysis of age-related changes in immune cell function and can provide a more holistic, systems-level view of immune cell signaling networks in the aging immune system. Recent work has demonstrated the utility of SCNP in identifying associations between immune cell signaling responses and age within distinct immune cell subsets in samples from healthy donors [7,8].

In the current study, SCNP was applied to perform a multi-dimensional analysis of intracellular immune signaling in an independent cohort of healthy donors consisting of 144 elderly donors and 30 young donors. Overall, 168 immune signaling responses (i.e. 24 signaling nodes, or combinations of modulator and intracellular readout, within 7 distinct immune cell subsets) were measured in PBMCs from the 174 healthy donors. Based on analyses of the previously published 60 donor cohort, 11 age-associated changes in cell signaling responses in CD45RA+ T cell subsets were pre-specified for testing in this current, independent study cohort and 9 of these age associations were verified. The confirmation of age associations in an independent donor cohort supports the use of the SCNP technology as a tool for identifying reproducible predictive biomarkers in areas such as vaccine response and response to cancer immunotherapies.

In a broader exploratory analysis of age-related immune signaling changes performed in the current donor cohort, 57 immune signaling responses (61% of the 93 responsive signaling nodes) were significantly associated with age. The age-associated signaling responses spanned all modulators (IFN-α, IFN-γ, IL-2, IL-4, IL-6, IL-10, IL-27, anti-IgD, LPS, R848, PMA, and CD40L) and all subsets (monocytes, B cells, CD3-CD20- lymphocytes (NK cell-enriched subpopulation), CD45RA+ Th cells, CD45RA- Th cells, CD45RA+ cytotoxic T cells, and CD45RA- cytotoxic T cells) demonstrating the importance of a holistic approach to describing immunosenescence.

## Methods

### Participants and PBMC samples

**Figure 1:**
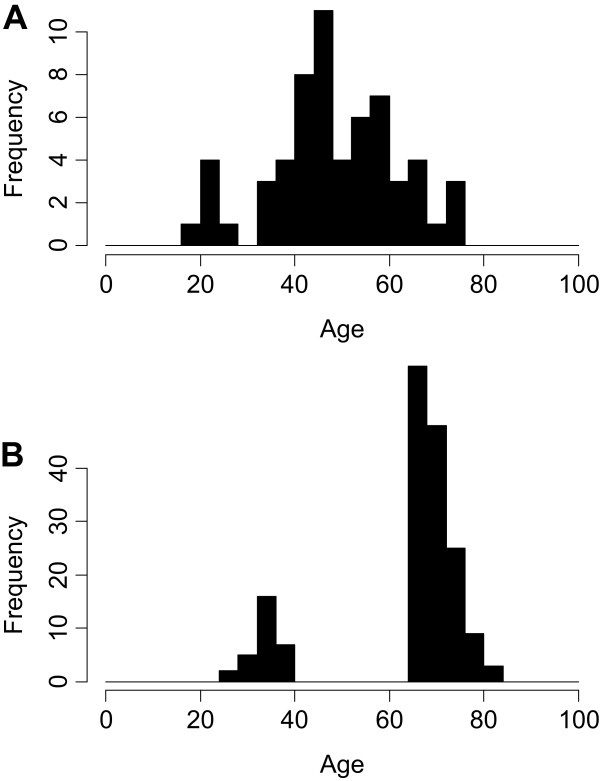
**Distributions of donor ages.** Histograms of donors’ ages in the previously published (training) donor cohort **(A)** and in the current study (test) donor cohort **(B)**.

The current study was registered with ClinicalTrials.gov (NCT01119703) and approved by an independent Canadian institutional review board. Participants were 174 healthy residents of Quebec who gave written informed consent prior to collection of blood samples. They were aged either ≥ 65 or 25–40 yrs (Table 1, Figure 1B), of European ancestry, and 55% female.

**Table 1:** Summary of donor numbers, age, and gender

	**Published study (Training set)**	**Current study (Test set)**
Number of donors	60	174
Median age (Range)	48 (19–73) yrs	68 (25–83) yrs
Gender	48 Male	78 Male
	12 Female	96 Female

Donor characteristics are shown for the previously published study (training set) and in the current study (test set) cohort. Age distributions for both cohorts are shown in Figure 1.

Blood was collected into sodium heparin Vacutainer® tubes (Becton-Dickinson) from which PBMCs were isolated and cooled within 8 h of collection. Briefly, heparinized blood was diluted 1:1 with RPMI-1640 medium and overlaid on ficoll/hypaque for centrifugation – all at room temperature. Resultant PBMC were washed twice with RPMI, resuspended in ice-cold 10% DMSO/90% heat-inactivated FBS at a concentration of 10 million cells per ml, and frozen slowly using a -1°C/min device to -80°C. Cells were placed into liquid nitrogen temperatures within 72 h of freezing and transported in a liquid nitrogen dry shipper. All samples had viabilities >70% based on cleaved poly (ADP-ribose) polymerase negativity.

The PBMC samples from the 60 healthy donors used for training purposes have been described previously [7]. Briefly, cryopreserved PBMC samples were collected from healthy donors within the Department of Transfusion Medicine, Clinical Center, National Institutes of Health, with institutional review board approval. All healthy donors donated blood samples for research purposes with informed consent.

### SCNP assay and reagents

The SCNP assay was performed as described previously [7]. Modulators and concentrations were as follows: 1000 IU/ml IFN-α (PBL); 50 ng/ml IL-4, 5 μg/ml anti-IgD (BD Biosciences); 50 ng/ml IL-2, 50 ng/ml IL-6, 50 ng/ml IL-27, 5 μg/ml R848 (Invivogen); 40 nM PMA (Sigma Aldrich), 3 μg/ml anti-CD3 (eBioscience), 10 μg/ml anti-mouse (Santa Cruz Biotechnology), 10 μg/ml anti-IgM (Southern Biotech). For TCR stimulation, cells were exposed to anti-CD3 for 12 min, with anti-mouse added for the last 2 min; the anti-IgM modulation time was 10 min; all other modulation times were 15 min. Staining was performed using Ab cocktails with each cocktail consisting of 5 Abs to detect phenotypic markers and 2 Abs to detect intracellular protein readouts. Abs used include anti-CD3, -CD4, -CD45RA, -CD20, -p-NF-κB, -c-poly(ADP-ribose) polymerase, -p-Stat1, -p-Stat3, -p-Stat5, -p-Stat6, -p-Erk, -p-ZAP70/Syk (BD Biosciences); -p-Akt, -p-S6 (CST); and -CD14 (Beckman Coulter). Flow cytometry data was acquired on FACS Canto II Flow Cytometers (BD Biosciences). All flow cytometry data were analyzed with WinList (Verity House Software). PBMC subpopulations were delineated according to the immunophenotypic gating scheme described previously [7].

### SCNP terminology and metrics

The term “signaling node” refers to a specific protein readout in the presence of a specific modulator. For example, the response to IFN-α stimulation can be measured using p-Stat5 as a readout. This signaling node is designated “IFN-α → p-Stat5”. Each signaling node is measured in each of the 7 distinct cell subpopulations simultaneously. The cell subpopulation is noted following the node e.g. “IFN-α → p-Stat5 | CD45RA- Th cells”. The raw instrument median fluorescence intensities (MFIs) were converted to calibrated intensity metrics, Equivalent Number of Reference Fluorophores (ERFs), by using rainbow calibration particles on each 96-well plate [9,10]. The “Fold” metric is applied to measure the level of a signaling molecule after modulation compared to its level in the unmodulated state.

The “Fold” metric was calculated as follows:

**Fold**: log_2_ [ERF(Modulated)/ERF(Unmodulated)]

Overall, 24 signaling nodes (Table 2), or modulated protein readouts, were measured in 12 cell populations defined by their surface phenotypes including 7 distinct immune cell subpopulations (monocytes, B cells, CD3-CD20- lymphocytes (NK cell-enriched subpopulation), CD45RA+ Th cells, CD45RA- Th cells, CD45RA+ cytotoxic T cells, and CD45RA- cytotoxic T cells) within unsorted PBMC samples from 174 healthy donors (Table 1). In the current study, Th cells and cytotoxic T cells are defined based on the differential expression of CD4 (i.e. CD4+ cells are categorized as Th cells and CD4- cells are categorized as cytotoxic T cells).

**Table 2:** Signaling nodes measured

**Signaling node**	**Measured in prior study**
IFN-α → p-Stat1	Yes
IFN-α → p-Stat3	Yes
IFN-α → p-Stat5	Yes
IFN-α → p-Stat6	Yes
IL-2 → p-Stat5	Yes
IL-2 → p-Stat6	Yes
IL-4 → p-Stat5	Yes
IL-4 → p-Stat6	Yes
IL-6 → p-Stat1	Yes
IL-6 → p-Stat3	Yes
IL-27 → p-Stat1	Yes
IL-27 → p-Stat3	Yes
IL-27 → p-Stat5	Yes
IL-27 → p-Stat6	Yes
R848 → p-Erk	Yes
R848 → p-NF-κB	Yes
PMA → p-S6	Yes
PMA → p-Erk	Yes
Anti-IgD → p-S6	No
Anti-IgD → p-Erk	No
Anti-IgM → p-ZAP70/p-Syk	No
Anti-IgM → p-Erk	No
Anti-CD3 → p-ZAP70/p-Syk	No
Anti-CD3 → p-Erk	No

The following 24 signaling nodes (modulator and intracellular readout combinations) were measured in the current study. The majority of these signaling nodes (i.e. the 18 signaling nodes indicated above) were also measured in the previously published study cohort [7]. All signaling nodes were measured in 12 cell populations defined by their surface phenotypes including 7 distinct immune cell subpopulations (monocytes, B cells, CD3-CD20- lymphocytes (NK cell-enriched subpopulation), CD45RA+ Th cells, CD45RA- Th cells, CD45RA+ cytotoxic T cells, and CD45RA- cytotoxic T cells).

### Statistical analysis

A train and test approach was utilized for verification of associations between immune signaling response and age in two independent healthy donor cohorts. Here, the 60 donor cohort (Table 1, Figure 1A) from a previously published study (i.e. the master set in the prior cohort [7]) was used as a training set, and associations between immune signaling responses and age were identified by regressing age against each signaling node in each distinct cell subset using a linear model. The *p* value corresponding to the hypothesis that the slope is zero was used as the significance test (with a *p* < 0.05 cutoff). Immune signaling responses with significant associations with age in the 60 donor training set were pre-specified for confirmation in the independent set of 174 healthy donors in the current study (test) cohort. To control the type I error rate, pre-specified age associations were ranked (based on the strength of the association with age in the training cohort) and tested using a Gatekeeper strategy [11]. In this strategy, each hypothesis to be verified in the test set must be prospectively specified and ordered, and subsequently tested in that order (here specified in Table 3). A hypothesis is only considered verified if it is significant in the test set and all other hypotheses tested prior to it are significant. In the test set, age associations were considered significant if *p* < 0.05 for the two-sided Wilcoxon rank-sum test statistic. The Wilcoxon rank-sum test was used for analyzing age associations in the current study (test) cohort because this cohort contained distinct young and elderly donor subgroups (Figure 1B). Because the previously published (training) cohort did not contain distinct donor subgroups based on age (as shown by the age distribution in Figure 1A), linear regression was used for analyzing age associations in that cohort as described above.

**Table 3:** Pre-specified hypotheses for testing in the current study cohort

**Hypothesis order**	**SCNP Node | Cell subset**	**Wilcoxon p-value**	**Hypothesis verified?**
**1**	IL-2 → p-Stat5 | CD45RA+ Th cells	1.10 × 10^-8^	Yes
**2**	IFN-α → p-Stat5 | CD45RA+ cytotoxic T cells	5.65 × 10^-10^	Yes
**3**	IL-27 → p-Stat5 | CD45RA+ cytotoxic T cells	9.38 × 10^-11^	Yes
**4**	IFN-α → p-Stat1 | CD45RA+ cytotoxic T cells	8.67 × 10^-8^	Yes
**5**	IL-6 → p-Stat1 | CD45RA+ cytotoxic T cells	2.46 × 10^-9^	Yes
**6**	IL-4 → p-Stat6 | CD45RA+ cytotoxic T cells	1.86 × 10^-4^	Yes
**7**	IL-6 → p-Stat3 | CD45RA+ cytotoxic T cells	6.70 × 10^-7^	Yes
**8**	IFN-α → p-Stat5 | CD45RA+ Th cells	5.28 × 10^-6^	Yes
**9**	IL-27 → p-Stat1 | CD45RA+ cytotoxic T cells	7.70 × 10^-8^	Yes
**10**	IFN-α → p-Stat3 | CD45RA+ cytotoxic T cells	0.180	No
**11**	PMA → p-S6 | CD45RA+ cytotoxic T cells	4.82 × 10^-6^	No*

*This age-association is significantly associated with age in the test cohort, but not verified according to the requirements of the gatekeeper approach. Hypotheses were sequentially ordered for testing using a Gatekeeper strategy. A *p* value < 0.05 for the two-sided Wilcoxon rank-sum test statistic was considered significant.

To discount variation in cell viability as a causative factor for signaling differences between young and elderly donor subgroups, differences between the proportions of viable cells were analyzed. No significant association between cell viability and age was observed (*p* = 0.237, Wilcoxon rank-sum test statistic).

## Results and Discussion

### Verification of pre-specified associations between immune signaling responses and age in independent healthy donor cohorts

**Figure 2:**
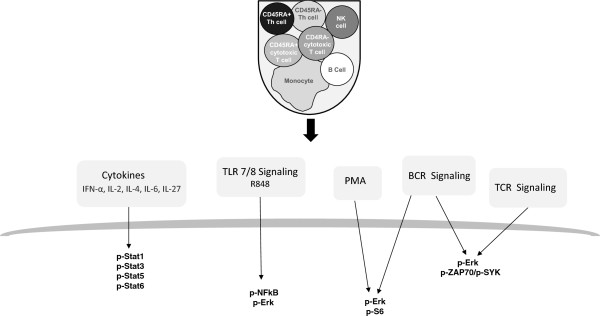
Diagram of the immune signaling pathways and cell subsets profiled in the current study cohort.

The master set of 60 donors from the published study [7] was utilized as a training data set and, among the panel of immune signaling responses profiled (Table 2) in the training cohort, responses with significant associations with age (based on linear regression analysis, see Methods) were identified. Eleven immune signaling responses (Table 3) that were significantly associated with age in the 60 donor training set were pre-specified for confirmation in the current study (test) cohort of 174 healthy donors (Table 1). Despite profiling a broad panel of signaling pathways across multiple immune cell subsets (Figure 2, Table 2), all of the immune signaling responses with significant associations with age in the 60 donor training cohort (i.e. the 11 pre-specified hypotheses), were within CD45RA+ T cell subsets. This result is consistent with reports that age-related defects in immune cell function are most pronounced within the naïve T cell compartment [12].

**Figure 3:**
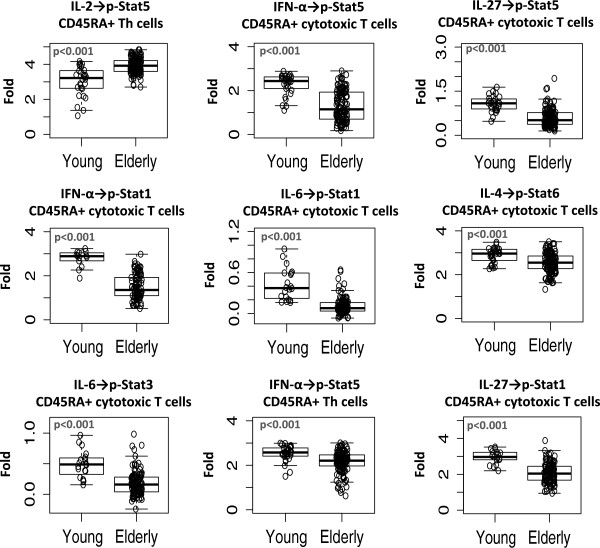
**Verified age-associated immune signaling responses.** Boxplots for Young and Elderly donors are shown for the 9 age-associated immune signaling responses that were independently verified in the current study cohort using the Gatekeeper strategy. The boxplots were constructed using the 1^st^ quartile, the median, and the 3^rd^ quartile. The whiskers represent the lowest and highest data points within 1.5 IQR (interquartile range). The circles represent data points for individual donors.

Using a Gatekeeper strategy (see Methods), 9 of the 11 pre-specified age-associated signaling responses were verified in the independent cohort of 174 donors (test set) in the current study. Figure 3 shows the range of signaling in the elderly vs. the young donors for the 9 verified age-associated responses. The verified age associations included 7 immune signaling responses in the CD45RA+ cytotoxic T cell subset. Specifically, in response to the cytokines IFN-α, IL-4, IL-6, and IL-27, the elderly donors had lower induced p-Stat levels in CD45RA+ cytotoxic T cells than the younger donors. An age-related decline in the responsiveness of the JAK-STAT signaling pathway within this cytotoxic T cell subset may contribute to age-related functional and phenotypic changes reported to occur in cytotoxic T cells [13].

Within the CD45RA+ Th cell compartment, the verified associations included age-associated differences in IFN-α-induced p-Stat5 and in IL-2-induced p-Stat5 responses. Notably, CD45RA+ Th cells displayed higher IL-2-induced p-Stat5 in elderly donors than in young donors. Because IL-2 production is reported to decrease with age [14], the heightened responsiveness to IL-2 in CD45RA+ Th cells observed in this study in the elderly might be considered a compensatory mechanism. Clinically, the use of IL-2 as an immunotherapy has shown promise in the treatment of cancers including renal cell carcinoma and malignant melanoma [15]. IL-2 immunotherapy may help to restore the proliferative capacity of naïve T cells in elderly cancer patients and may be a particularly effective approach for augmenting the effect of checkpoint-related immunotherapies. However, the effectiveness of IL-2 immunotherapy may be limited if, as suggested previously, the induction of p-Stat5 via IL-2 is not directly coupled with proliferative responses due to the presence of age-related defects in responsiveness downstream of p-Stat5 induction [16].

### Age-associated immune responses identified within the current study cohort span all signaling pathways and immune cell subsets profiled

**Figure 4:**
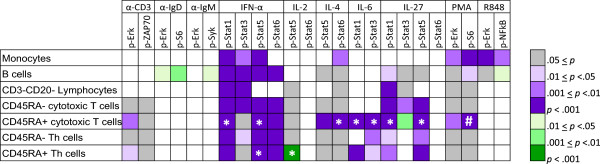
**Age-associated immune signaling responses in the current study cohort.** Wilcoxon (two-sided) test statistics were calculated for each node in each cell subpopulation. Responses that differed significantly between Young and Elderly donors are indicated with purple (lower responses in Elderly than Young) and green (higher responses in Elderly than Young) with the color intensity corresponding to the magnitude of the *p* value. Asterisks indicate the 9 responses verified using the Gatekeeper approach. The number sign indicates the pre-specified response significantly associated with age that was not verified by the Gatekeeper approach. Solid gray boxes represent nodes that were responsive in the given cell subset but did not associate with age (*p* > 0.05). Solid white boxes represent nodes that did not show a response in the corresponding cell subset.

Age-associated alterations in immune signaling responses were further explored within the current study cohort by identifying all responses among the broad panel of immunomodulators and immune cell subsets profiled (Figure 2, Table 2) which differed significantly between young and elderly donors. As shown in Figure 4, across the 168 immune signaling responses measured (i.e. 24 signaling nodes in 7 distinct cell subsets), 93 were responsive (based on a Fold threshold of 0.25 representing an ~1.2-fold change in modulated levels relative to basal, see Methods). The observed cell subset-specificity in responsiveness was consistent with prior studies [7] and expected based on reported cell-subset specific expression of receptors for the ligands included in the study. For example, for the TLR7/8 agonist, R848, the observed responsiveness of the R848 → p-Erk and R848 → p-NF-κB signaling nodes in monocytes and B cells but not in T cell subsets (as indicated by the white boxes in Figure 4) is consistent with the cell-subset specific expression of the receptor (TLR7/8) on monocytes and B cells.

Of the 93 responsive signaling nodes, 57 showed significant age-associated differences with the majority (51 nodes) displaying lower responsiveness in elderly donors than in younger donors (purple boxes in Figure 4). Targeted therapeutic re-activation of these signaling responses may allow for restoration of immune function in the elderly. Only 6 immune signaling responses had a higher magnitude in elderly donors than in the younger donors (green boxes in Figure 4). These 6 responses include one of the verified hypotheses (IL-2 → p-Stat5 | CD45RA+ Th cells) discussed above, IL-27 → p-Stat3 | CD45RA+ cytotoxic T cells, and 4 signaling nodes (anti-IgD → p-Erk, anti-IgD → p-S6, anti-IgM → p-Syk, and R848 → p-NF-κB) in B cells. Because memory B cells and naïve B cells differ in their responsiveness to many stimuli [17], the higher responsiveness observed here for 4 signaling nodes in the overall parent B cell population in elderly donors compared to younger donors may be reflective of the reported age-related decline in memory B cells [18]. The hypothesis that the higher signaling in elderly than in younger donors following modulation with anti-IgD and anti-IgM is related to a difference in proportion of B cell subsets is further supported by previous work which has demonstrated that there is a positive relationship between the frequency of IgD + B cells and the magnitude of BCR signaling responses detected in the parent B cell population [19]. Future studies that include phenotypic markers such as CD27 and IgD to delineate B cell subpopulations are needed to investigate age-related differences in immune signaling responses within distinct B cell subpopulations.As noted above, the verified age-associated responses (which differed significantly with age in both the current study cohort and the previously published cohort) were restricted to cytokine signaling responses in CD45RA+ T cell subsets (indicated by asterisks in Figure 4). In contrast, the 57 significant age-associated responses identified within the current study cohort spanned all modulators and all 7 distinct immune cell subsets profiled in the 174 healthy donors. Notably, all of the responsive signaling nodes in monocytes displayed significant associations with age. Among the four T cell subsets, 50% (both CD45RA- subsets) to 81% (CD45RA+ cytotoxic T cells) of the responsive signaling nodes displayed significant age associations. The NK-enriched cell subset appears to be the least impacted by age, with 38% of the responsive signaling nodes within this subset showing an association with age. Among the different immunomodulators profiled, differences in the effect of age on responsiveness were observed with some stimuli (i.e. IL-6, IL-27, and IFN-α) displaying significant associations with age for the majority of the responsive intracellular readouts measured and other immunomodulators (i.e. α-CD3 and IL-4) having a relatively small percentage (25%) of responsive intracellular readouts showing an association with age. Overall, these results suggest that age-related alterations in immune cell function are widespread, impacting multiple immune cell types and immune signaling pathways, with some degree of cell-subset specificity and stimulus-specificity in the sensitivity to age-related changes.

In the exploratory analysis described here, because the focus was on hypothesis generation, the less stringent approach of utilizing *p* values that do not account for multiple testing was employed. However, *p* values were subsequently adjusted for multiple testing using the Benjamini-Hochberg method to assess which age associations remain significant with this more conservative approach. Of the 57 age-associated signaling responses with (unadjusted) *p* values < 0.05, 47 have adjusted *p* values < 0.05. The 10 responses that lose significance with the adjusted *p* values include the 9 responses in Figure 4 with 0.01 < *p* < 0.05 and 1 response with *p* = 0.007 (IL-27 → p-Stat3 in CD45RA-cytotoxic T cells).

While a small number of the age-associated responses identified in the current study were not profiled in the previously published healthy donor cohort (Table 2), the majority of responses were measured in both donor cohorts. Age associations which reached statistical significance in the current study cohort may have failed to reach significance in the prior cohort for several possible reasons: 1) there was lower power for detecting significant differences in the prior study than in the current study due to sample size differences (Table 1), 2) age-associated differences may be more pronounced within a cohort containing only young (<40 yrs) and elderly (>65 yrs) donors than in a cohort in which the majority of the donors are between 40–65 yrs (Figure 1), or 3) some of the age-associations identified within the current cohort could be “false positives”. While future studies are needed to confirm many of the age associations identified by SCNP in the larger 174 healthy donor cohort, other groups have previously reported a subset of these age-associated alterations.

For example, an age-associated alteration in TCR signaling (a pathway which was not profiled in the prior study, see Table 2) is consistent with published reports [20]. In fact, recent work has demonstrated that there is an age-associated decline in TCR-induced Erk phosphorylation in naïve Th cells, but no age associated difference in other TCR-induced responses including ZAP70 in naïve Th cells, and no age-associated difference for TCR-induced responses in memory Th cells [21]. The results of the current study are remarkably consistent with these previously published reports [i.e. as shown in Figure 4, 1) there is a statistically significant association with age for TCR-induced p-Erk in CD45RA+ T cell subsets (predominantly naïve T cells) but not in CD45RA- memory T cell subsets and 2) TCR-induced p-ZAP70 is not significantly associated with age in any of the T cell subsets]. By integrating TCR signaling data with transcript expression measurements, Li et al. have recently demonstrated that the age-associated decline in TCR-induced Erk phosphorylation in naïve Th cells is due to an age-related increase in the phosphatase DUSP6 which is in turn a result of an age-related decline in the expression of miR-181a expression [21]. In the present study, the T cell subsets simultaneously profiled included not only the Th cell subsets studied by Li et al. but also CD45RA+/-cytotoxic T cell subsets, which similarly displayed a significant age-associated decline in TCR-induced Erk phosphorylation specifically within the CD45RA+ cytotoxic T cell compartment (Figure 4), suggesting that a decline in miR-181a expression and thus DUSP6 activity may affect immune signaling in an age-associated manner in the naïve cytotoxic T cell subset as well.

The age-associated decline in intracellular signaling responses in monocytes following modulation with the TLR7/8 ligand R848 (Figure 4) is consistent with published reports describing age-related alterations in TLR responses. Specifically, van Duin et al. have reported reduced TLR-induced CD80 expression on monocytes from older donors compared to younger donors [22]. In our SCNP studies with a small number of PBMC samples from healthy control donors, the magnitude of intracellular signaling responses following short-term (15 min) TLR modulation has shown a positive correlation with increased CD80 and CD86 expression following long-term (~21 hr) modulation with the same TLR ligands (data not shown).

Future work will focus on expanding the immune cell subsets interrogated for age-associated signaling alterations. The inclusion of phenotypic markers allowing for the delineation of additional cell subsets, such as dendritic cells, will allow for a greater understanding of age-related alterations in innate immunity [23]. Subsequent studies including additional surface markers will also enable the analysis of increasingly homogeneous cell subsets within the broader phenotypes described herein. For example, the CD45RA+ cytotoxic T cell subset described in the current study can be further subdivided into naïve and effector cell subsets via the inclusion of markers such as CD27, CD28, or CCR7. Because the composition of the more heterogeneous CD45RA+ cytotoxic T cell subset may change with age, it will be important to analyze age-associated differences in immune signaling responses in increasingly homogeneous cell subsets, building upon the information obtained in the current study. The incorporation of multiple time points following modulation will further enable an analysis of age-associated differences in signaling kinetics. Finally, analyses will integrate SCNP intracellular signaling data with additional biomarkers generated from gene expression studies, cytokine production analysis, and sequencing measurements.

## Conclusions

In summary, SCNP provides quantitative, high dimensional measurements of the human immune cell signaling network pertinent to unraveling the complex mechanisms underlying the age-related decline in the function of the human immune system. The confirmation of age associations in two independent donor cohorts supports the utility of SCNP as a tool for identifying associations between immune signaling responses and clinical outcomes such as response to classical prophylactic vaccines, therapeutic cancer vaccines, or alternative immunotherapies.

## Abbreviations

SCNP: Single-cell network profiling.

## Competing interests

DML., BL, JP, GF, EE, SP, MA, DS, AC, and REH are employees of Nodality Inc. AS, SL, RR, CRB, and LNC are employees of Merck Inc. The other authors declare no financial conflicts of interest.

## Authors’ contributions

DML, REH, AC, CRB, and LNC conceived the study. AS and MA coordinated clinical sample acquisition and sample handling. DML. performed the experiments. DML, BL, and EE analyzed the experiments. JP, GF, SP, DS, and RR participated in the review of the study analysis. DML. drafted the manuscript. All authors read and approved the final manuscript.
